# Supplementation with sunflower or soybean oil: ruminal parameters and fatty acid profile in a Rusitec system

**DOI:** 10.29374/2527-2179.bjvm009026

**Published:** 2026-11-10

**Authors:** Gabriela Casarotto, Alejandro Britos, Ignacio Vieitez, Cecilia Cajarville, Silvana Carro

**Affiliations:** 1 Departamento de Ciencia y Tecnología de los Alimentos, Facultad de Veterinaria, Universidad de la República, Montevideo, Uruguay; 2 Departamento de Producción Animal y Salud de los Sistemas Productivos, Facultad de Veterinaria, Universidad de la República, Libertad, San José, Uruguay; 3 Departamento de Ciencia y Tecnología de los Alimentos, Facultad de Química, Universidad de la República, Montevideo, Uruguay

**Keywords:** biohydrogenation, dietary oil, rumen environment, biohidrogenação, óleos alimentares, ambiente ruminal

## Abstract

The current worldwide demand for healthier animal-derived foods has led to modifications in ruminant diets aimed at improving product composition, particularly the fatty acid profile. Supplementing dairy cow diets with vegetable oils may be an effective strategy to improve fatty acid composition. This study evaluated the inclusion of two vegetable oils with different fatty acid profiles in a high-quality forage-based diet. Five diets were incubated in a Rusitec system: a basal diet (C) containing alfalfa and a commercial concentrate (65:35, DM basis), and the same diet supplemented with sunflower oil (SF) or soybean oil (SB) to reach 60 g/kg DM (SF60 and SB60) and 75 g/kg DM (SF75 and SB75). After a 5-day adaptation period, fermentation parameters, nutrient degradability, and the ruminal fatty acid profile were evaluated. Oil supplementation did not affect ruminal pH, gas production, total volatile fatty acid concentration, or nutrient degradability. All supplemented treatments increased trans-vaccenic acid (C18:1 *t*11, TVA) by approximately 42–47% compared with the control diet (P<0.001). In addition, dietary treatment affected oleic acid (C18:1 *c*9), monounsaturated fatty acids (MUFA), and saturated fatty acids (SFA), with higher oleic acid and MUFA concentrations observed in SF75, SB60, and SB75 than in the control, whereas SFA concentrations were lower in SF75 and SB60. These results indicate that supplementation with sunflower or soybean oil at levels up to 75 g/kg DM does not negatively affect ruminal fermentation or nutrient disappearance *in vitro*, indicating that both oils are promising dietary strategies for improving the ruminal fatty acid profile.

## Resumo

A crescente demanda mundial por alimentos de origem animal mais saudáveis para o consumo humano tem levado à adoção de mudanças na alimentação de ruminantes, visando melhorar principalmente o perfil lipídico desses produtos. Nesse contexto, a suplementação das dietas de vacas leiteiras com óleos vegetais pode ser uma estratégia eficaz para alterar a composição dos ácidos graxos. Assim, este estudo teve como objetivo avaliar a inclusão de dois óleos vegetais com diferentes perfis de ácidos graxos em uma dieta baseada em forragem de alta qualidade. Foram incubadas cinco dietas em um sistema Rusitec: uma dieta controle (C), composta por alfafa e um concentrado comercial (65:35, com base na MS), e a mesma dieta suplementada com óleo de girassol (SF) e óleo de soja (SB), em níveis de 60 g/kg de MS (SF60 e SB60) e 75 g/kg de MS (SF75 e SB75). Após um período de adaptação de cinco dias, foram analisados os parâmetros de fermentação, a desaparição dos nutrientes e o perfil de ácidos graxos do fluido ruminal. A suplementação com óleo não afetou o pH ruminal, a produção de gás, a concentração total de ácidos graxos voláteis ou o desaparecimento dos nutrientes. Todos os tratamentos suplementados aumentaram a concentração do ácido trans-vaccênico (C18:1 *t*11, TVA) em aproximadamente 42–47% em comparação com a dieta controle (P<0,001). Além disso, a suplementação com óleo afetou as concentrações de ácido oleico (C18:1 *c*9), ácidos graxos monoinsaturados e ácidos graxos saturados, com maiores concentrações de ácido oleico e MUFA nos tratamentos SF75, SB60 e SB75 em comparação com o controle, enquanto as concentrações de SFA foram menores nos tratamentos SF75 e SB60. Esses resultados indicam que a suplementação com óleo de girassol ou soja em níveis de até 75 g/kg de MS não afeta negativamente a fermentação ruminal ou o desaparecimento de nutrientes in vitro, indicando que ambos os óleos são estratégias dietéticas promissoras para melhorar o perfil de ácidos graxos ruminais.

## Introduction

In ruminant-derived dairy and meat products, there is considerable interest in increasing the concentration of conjugated linoleic acid (CLA), a group of fatty acid isomers with 18 C atoms and two conjugated double bonds, widely studied for their potential anticarcinogenic and antiatherogenic properties (Koba & Yanagita, 2013; Pariza & Ha, 1990; Woods & Fearon, 2009). This group is mainly synthesized in the rumen as an intermediate of the biohydrogenation (BH) of dietary linoleic acid (LA, C18:2 *c*9, *c*12) (Bauman et al., 2000), before being fully converted into stearic acid (C18:0). The main CLA isomer, rumenic acid (C18:2 *c*9, *t*11, is formed in the rumen during LA biohydrogenation, with *Butyrivibrio fibrisolvens* participating in this process (Kim et al., 2000), and can also be synthesized in the mammary gland from trans-vaccenic acid (TVA, C18:1 *t*11) via $\Delta^{9}$-desaturase (Doreau et al., 2016; Maia et al., 2010). Another fatty acid that contributes to CLA formation is α-linolenic acid (ALA), which comes from the diet and is converted to TVA in the rumen before being fully saturated (Doreau et al., 2016).

Ruminant supplementation with oils, such as soybean and sunflower oils, is a common strategy to increase energy density and improve the fatty acid profile of milk (Dauber et al., 2021; Vargas et al., 2023). These oils are important dietary sources of unsaturated fatty acids, particularly LA, and can effectively increase CLA levels in milk (Dauber et al., 2022; Matsushita et al., 2007). However, oil supplementation can impair the ruminal microbial community, either through its direct toxicity to microorganisms or by inhibiting microbial colonization and enzyme activity (Vargas et al., 2019). This negative effect is more pronounced when the level of oil increases, particularly with oils containing high LA and ALA concentrations (Kim et al., 2000).

The existing literature on soybean and sunflower oil supplementation has largely concentrated on their effects on the fatty acid composition of final products such as milk and meat. However, fewer studies have focused on how these oils are transformed within the rumen or on their impact on nutrient digestibility (Kokic et al., 2024). Moreover, in most studies, oils have been supplemented in the context of high-concentrate diets. Supplementing high-forage diets with vegetable oils could be particularly advantageous in regions where grazing is a common management practice, such as in many parts of the southern hemisphere.

Understanding the ruminal metabolism of dietary lipids is essential for predicting the extent to which supplemented oils contribute to the final fatty acid profile of ruminant-derived products. The biohydrogenation of unsaturated fatty acids in the rumen is a complex microbial process that determines the flow of bioactive intermediates, such as vaccenic and rumenic acids, to the duodenum (Jenkins et al., 2008). *In vitro* fermentation systems, particularly the Rusitec system, offer a controlled and reproducible approach to examine these transformations without the confounding effects of animal variability, endogenous synthesis, or post-ruminal absorption (Hristov et al., 2012). Such systems are especially valuable for isolating the specific effects of oil supplementation on rumen fermentation parameters, nutrient disappearance, and the accumulation of fatty acid intermediates. However, to the best of our knowledge, no studies to date have specifically examined the ruminal transformation of sunflower and soybean oils when added to high-forage diets using the Rusitec system. This is particularly relevant in regions where grazing is a common management practice, such as many parts of the southern hemisphere, as the rumen environment and microbial populations can differ substantially between high-forage and high-concentrate diets, potentially altering biohydrogenation pathways and the resulting fatty acid profile available for incorporation into milk and meat.

The present study aimed to address this gap by evaluating the inclusion of sunflower and soybean oils at two concentrations in a high-quality forage-based diet incubated in a Rusitec system. We hypothesized that supplementation with sunflower or soybean oil would increase the accumulation of biohydrogenation intermediates, such as CLA and TVA, depending on the level of oil supplementation, without negatively affecting ruminal fermentation characteristics.

## Materials and methods

All procedures were approved by the Animal Use Ethics Committee of Facultad de Veterinaria. The experiment was conducted at the Rumen Fermentation Laboratory, Facultad de Veterinaria, UdelaR, Uruguay (34°S 55°W).

### Experimental diets

An experimental diet was formulated according to National Academies of Sciences, Engineering, and Medicine (2021) for a cow producing 30 kg/day of milk and consuming 20 kg of DM/day of a total mixed ration with a forage:concentrate ratio of 65:35 (Table 1). Alfalfa hay (*Medicago sativa*) was used as forage, and the included concentrates were corn grain, wheat bran, soybean meal, calcium carbonate, sodium chloride, monocalcium phosphate, and a vitamin-mineral premix. This diet was used as the control diet (C), and the other treatments consisted of adding soybean oil (SB) or sunflower oil (SF), each at two doses: until reaching 60 g/kg of DM (60) and until 75 g/kg DM (75) of the whole diet. Therefore, five treatments were established: C, SF60, SF75, SB60, and SB75 (Table 1). The inclusion levels of 60 and 75 g/kg DM were selected to represent moderate and high lipid supplementation levels commonly used in previous studies. The 60 g/kg DM level is consistent with previous in vitro studies (Jacob et al., 2012; Vargas et al., 2019), whereas the 75 g/kg DM was included to investigate whether a higher supply of unsaturated fatty acids could further modify ruminal biohydrogenation without negatively affecting fermentation and nutrient degradability.

**Table 1:** Ingredients, chemical composition, and fatty acid profile of the experimental diets (g/kg DM, if no other basis is specified).

	**C**	**SF60**	**SF75**	**SB60**	**SB75**
**Ingredients**					
Alfalfa hay	650	631	621	631	621
Corn grain	132	128	126	128	126
Wheat bran	132	128	126	128	126
Soybean meal	66	64	63	64	63
Calcium carbonate	7	6	6	6	6
Sodium chloride	8	8	8	8	8
Monocalcium phosphate	3	3	3	3	3
Vitamin mineral premix	2	2	2	2	2
Soybean oil	-	-	-	30	45
Sunflower oil	-	30	45	-	-
**Chemical composition**					
DM, g/kg FM	863	869	871	867	870
OM	905	900	907	902	907
CP	189	177	181	179	176
EE	29	58	75	55	73
aNDF	339	335	299	334	334
ADF	213	225	206	227	204
**Fatty acid composition, g/100g fat**					
C16:0	26.78	11.52	10.76	17.33	16.65
C18:0	3.42	4.32	4.32	6.09	5.46
C18:1 *c*9	29.61	40.73	43.73	26.28	33.22
C18:2 *c*9*, c*12	32.92	39.95	38.54	43.80	39.92
C18:3 *c*9*, c*12*, c*15	3.81	1.25	1.13	4.15	3.37
C20:1 *c*11	0.57	0.41	0.33	0.94	0.38
Total SFA	30.20	15.84	15.08	23.42	22.11
Total MUFA	30.18	41.14	44.06	27.22	33.60
Total PUFA	36.73	41.20	39.67	47.95	43.29

C: control diet; SF60: control diet with sunflower oil added until reaching 60 g/kg EE of diet DM; SF75: control diet with sunflower oil added until reaching 75 g/kg EE of diet DM; SB60: control diet with soybean oil added until reaching 60 g/kg EE of diet DM; SB75: control diet with soybean oil added until reaching 75 g/kg EE of diet DM; DM: dry matter; FM: fresh matter; OM: organic matter; CP: crude protein; EE: ether extract; aNDF: neutral detergent fibre treated with amylase; ADF: acid detergent fibre; C16:0: palmitic acid, C18:0: stearic acid, C18:1 *c9*: oleic acid, C18:2 *c9, c12*: linoleic acid, C18:3 *c9, c12, c15*: α-linolenic acid, C20:1 *c11*: gondoic acid. Total SFA, total MUFA and total PUFA were calculated from the individual fatty acids presented in the table.

### Experimental design and in vitro fermentation procedure

The in vitro fermentation was conducted using a Rusitec system (Czerkawski & Breckenridge, 1977) equipped with six fermentation vessels, each with a capacity of 900 mL. Five of the six available fermenters were used in the present study, allowing one fermenter to be assigned to each dietary treatment during every experimental run. The experiment was conducted as a randomized complete block design with three independent experimental runs used as blocks. Five dietary treatments (C, SF60, SF75, SB60, and SB75) were evaluated in each run, with one fermenter assigned to each treatment. Each experimental run lasted 10 days, consisting of a 5-day adaptation period followed by a 5-day sampling period. Treatments were randomly assigned to the five fermenters at the beginning of each run so that every treatment occupied a different fermenter position across runs (Figure 1). Consequently, each dietary treatment was evaluated once per run, resulting in three independent experimental units per treatment.

**Figure 1:**
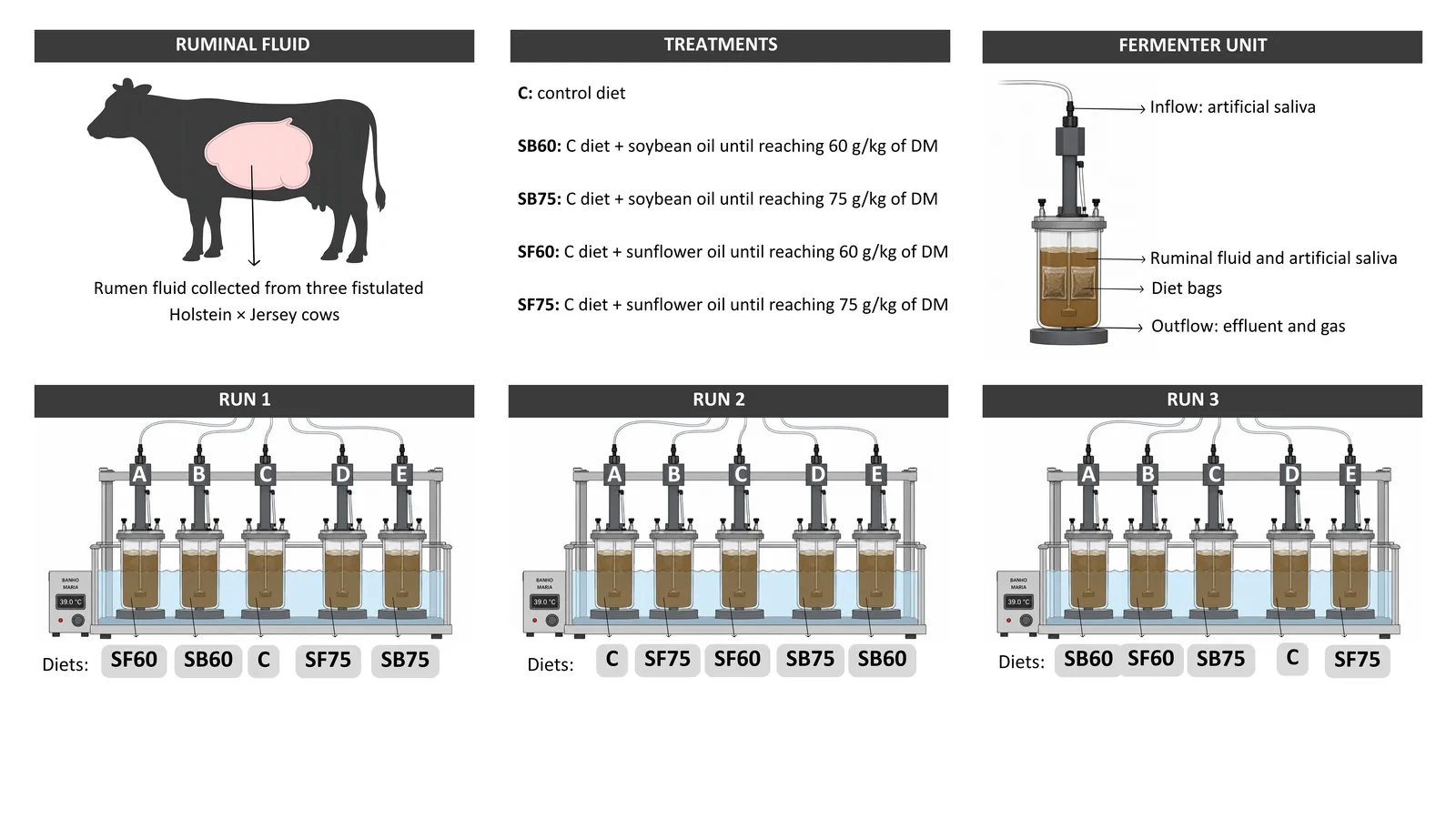
Schematic representation of the experimental design of the Rusitec study, showing the randomized allocation of the five dietary treatments among fermenters during each of the three independent experimental runs. Each run included one fermenter per treatment, resulting in three independent experimental units per treatment.

The inoculum (ruminal fluid) was collected from two fistulated Holstein × Jersey cows (550 ± 25 kg of body weight; 6 years old; non-lactating cows with no reproductive history) fed a diet consisting of 750 g/kg of alfalfa hay and 250 g/kg concentrate (125 g/kg corn grain + 125 g/kg soybean meal), approximately 2 hours after the morning feeding. Immediately after collection, the ruminal contents were filtered through four layers of cheesecloth to separate the solid and liquid fractions and transported to the laboratory in thermal containers within 10 minutes.

Approximately 780 mL of ruminal fluid was added to each fermenter together with two porous nylon bags (R510, ANKOM Technology Corp., USA): one containing 10 g of solid ruminal digesta and another containing 8 g DM of the respective experimental diet. Before filling the bags, the experimental diets were dried at 60°C and ground through a 2 mm sieve. After the first 24 hours of incubation, the bag containing solid rumen material was replaced with a bag containing the experimental diet, which remained in the fermenter for 48 h, with daily replacements.

The fermenters were immersed in a water bath maintained at 39°C, with continuous infusion of artificial saliva (McDougall, 1948) delivered through a peristaltic pump at a rate of 3% per hour, prepared daily. Diet bags were replaced daily at 1:00 pm, the gas produced was collected daily in hermetic bags (2 L capacity) with one-way valves, and the effluent was collected in 2 L Kitasato flasks located in a cold chamber to avoid microbial growth.

### Sample collection and laboratory analysis

During the 5-day sampling period, bags were removed from the fermentation unit, washed with artificial saliva, and stored at -20°C for analysis. Dry matter (DM) disappearance was determined for each bag, while a pooled sample for each treatment and run was used for crude protein (CP), neutral detergent fiber (NDF), and acid detergent fiber (ADF) disappearance. The pH was measured daily using a digital pH meter (Oakton® eChem Instruments Pte. Ltd., Singapore). Samples were also collected for volatile fatty acid (VFA) determination, ammonia nitrogen (${\text{NH}}_{3}\text{-N}$) analysis, and determination of the ruminal fluid fatty acid profile, with all samples stored at -20°C until analysis.

DM, CP, aNDF, and ADF disappearances (dDM,dCP,daNDF, and dADF) were determined from days 6 to 10. Prior to analysis, the bags were thawed, washed, dried, and weighed before grinding. DM, ash, and CP were analyzed according to Association of Official Analytical Chemists (1990), using methods 934.01, 942.05, and 984.13, while NDF and ADF were analyzed using an ANKOM220 fiber analyzer (ANKOM Technology Corp., USA) according to Robertson and van Soest (1981), using a nonsequential procedure with thermostable amylase. No sodium sulfite was used for aNDF analysis. Both fractions were expressed without residual ash content. Organic matter (OM) was calculated by difference from the ash content.

${\text{NH}}_{3}\text{-N}$ concentration was determined using the colorimetric method (Weatherburn, 1967), and VFAs were determined by high-performance liquid chromatography (HPLC) using a Dionex Ultimate® 3000 system at a wavelength of 210 nm. A 300 × 7.8 mm column (Acclaim, Rezex ROA-Organic Acid ${\text{H}}^{+}$ (8%), Phenomenex, USA) was used, following the method described by Adams et al. (1984). Fat was extracted from ruminal samples using the Röse-Gottlieb method (Association of Official Analytical Chemists, 1990). Fatty acid methyl esters were prepared according to the IUPAC 2.301 protocol (International Union of Pure and Applied Chemistry, 1987) and analyzed by gas chromatography (GC) using a Shimadzu (Kyoto, Japan) model 14B equipped with a Supelco (Bellefonte, PA) SP 2560 (100m×0.25mm×0.2 μm) capillary column and a flame ionization detector (FID). The injection volume of the samples was 1 μL. The temperature program was as follows: initial temperature 90°C for 2 min, increased to 175°C at 20°C/min and maintained for 35 min, then increased to 240°C at 15°C/min and maintained for 25 min. Peak identification was accomplished through the analysis of authentic standards. Standards and reagents used for the analysis were supplied by Sigma-Aldrich (United States).

### Statistical analysis

Data were analyzed using SAS OnDemand (SAS Institute Inc., Cary, NC, USA). Variables measured repeatedly over time (pH, gas production, ${\text{NH}}_{3}\text{-N}$ concentration, VFA concentration, dDM, and ruminal FA composition) were analyzed as repeated measures according to the following model:


$$Y_{ijkl}=\mu +T_{i}+D_{j}+{\left(T\times D\right)}_{ij}+B_{k}+\varepsilon_{ijkl}$$ (1)

Where Y*ijkl* is the dependent variable, 𝜇 is the general mean, T*i* is the fixed effect of the treatment (*i* = 1, …, 5), D*j* is the fixed effect of the sampling day (*j* = 1, …, 5), (T×D)*ij* is the interaction of treatment × day, B*k* is the random effect of the block (experimental run; *k* = 1, …, 3), and 𝛆*ijkl* is the residual error.

The individual fermenter was considered the experimental unit. Repeated measurements obtained from the same fermenter over consecutive sampling days were modeled using the REPEATED statement, with fermenter within experimental run specified as the subject. The covariance structure was selected separately for each response variable based primarily on the lowest Akaike Information Criterion (AIC), with the Bayesian Information Criterion (BIC) also considered. The covariance structure selected for each response variable is reported in the corresponding tables.

Variables without repeated measurements over time (dCP,daNDF, and dADF) were analyzed using PROC MIXED according to the following model:


$$Yik=\mu +Ti+Bk+\varepsilon_{ik}$$ (2)

Where Y*ik* is the dependent variable, 𝜇 is the general mean, T*i* is the fixed effect of the treatment, B*k* is the random effect of the block, and 𝛆*ik* is the residual error.

Residuals from all mixed models were evaluated for normality using the Shapiro–Wilk test (PROC UNIVARIATE). Variables that did not meet the assumption of normality were analyzed using generalized linear mixed models (PROC GLIMMIX) assuming a gamma distribution with a log link. Covariance structures were compared using the same procedure described for repeated mixed models. For variables analyzed using PROC GLIMMIX, statistical inference was performed on the model scale, whereas least-squares means and 95% confidence intervals are presented on the original scale using the inverse-link transformation to facilitate biological interpretation.

Least-squares means were compared using the Tukey–Kramer adjustment. Differences were considered significant at P<0.05, and trends were declared at 0.05≤P<0.10.

## Results

In this study, no significant differences were observed among treatments for ruminal pH, gas production, total VFA concentration, or nutrient disappearance (dDM, dCP, daNDF, and dADF; Table 2). No treatment × day interactions were detected for the repeatedly measured fermentation and disappearance variables (P>0.05).

**Table 2:** Rumen fermentation parameters, nutrient disappearance, and volatile fatty acid (VFA) concentration of the treatment diets in a Rusitec system.

	**Treatments**					**P-value**			
	**C**	**SF60**	**SF75**	**SB60**	**SB75**	**SEM**	**Treat.**	**Day**	**Treat. × Day**
pH	6.97	6.97	6.95	6.95	6.98	0.119	0.704	**0.014**	0.958
Gas, mL/d	644.4	683.1	696.2	657.4	619.6	42.346	0.615	0.206	0.457
NH_3_-N_,_ mg/dL	8.13^ab^	7.95^b^	9.78^a^	8.31^ab^	8.39^ab^	1.107	**0.031**	0**.058**	0.765
Total VFA, mM	35.61	35.07	37.87	35.10	36.09	1.611	0.735	0.913	0.698
VFA proportion, mol/100 mol									
Acetic	56.55	55.75	55.71	55.85	55.00	0.696	0.554	0.144	0.576
Propionic	34.22	34.85	34.57	34.82	35.63	1.442	0.968	**0.041**	0.968
Butyric	9.23	9.26	9.72	9.33	9.37	1.142	0.570	**0.082**	0.523
**Nutrient disappearance, proportion**									
dDM	0.532	0.564	0.561	0.562	0.553	0.016	0.479	0.275	0.614
dCP	0.598	0.609	0.621	0.617	0.610	0.027	0.891		
daNDF	0.230	0.262	0.234	0.250	0.261	0.022	0.695		
dADF	0.161	0.205	0.193	0.210	0.183	0.024	0.597		

C: control diet; SF60: control diet with sunflower oil added until reaching 60 g/kg EE of diet DM; SF75: control diet with sunflower oil added until reaching 75 g/kg EE of diet DM; SB60: control diet with soybean oil added until reaching 60 g/kg EE of diet DM; SB75: control diet with soybean oil added until reaching 75 g/kg EE of diet DM; ${\text{NH}}_{3}\text{-N}$: ammonia-N; Total VFA: sum of acetic, propionic, and butyric acid; dDM: dry matter disappearance; dCP: crude protein disappearance; daNDF: neutral detergent fiber treated with amylase disappearance; dADF: acid detergent fiber disappearance. Values are presented as least-squares means. Means within a row with different superscript letters differ significantly (P<0.05; Tukey–Kramer adjustment). Covariance structures used for repeated measurements were ARH(1) for pH and dDM, AR(1) for gas production, VC for ${\text{NH}}_{3}\text{-N}$ and total VFA, and UN for acetate, propionate, and butyrate.

Dietary treatment affected ruminal ${\text{NH}}_{3}\text{-N}$ concentration (P=0.031). The SF75 treatment had a higher concentration than SF60, whereas all other treatments showed intermediate values.

Sampling day affected ruminal pH (P=0.014) and the molar proportion of propionate (P=0.041), whereas a tendency for a day effect was observed for ${\text{NH}}_{3}\text{-N}$ concentration (P=0.058) and the molar proportion of butyrate (P=0.082).

The ruminal fatty acid composition is presented in Table 3. Dietary treatments affected the concentrations of *trans*-vaccenic acid (C18:1 *t*11, TVA; *P* < 0.001), oleic acid (C18:1 c9; *P* = 0.014), SFA (*P* = 0.026), and MUFA (*P* = 0.014). A tendency for a treatment effect was observed for palmitic acid (C16:0; *P* = 0.073).

**Table 3:** Ruminal fatty acid composition (g/100g fat) of the RUSITEC liquor after using different diets.

	**Treatments**					**SEM**	**P-value**		
	**C**	**SF60**	**SF75**	**SB60**	**SB75**		**Treat**	**Day**	**Treat. × Day**
C10:0	0.372	0.439	0.488	0.622	0.577	0.156	0.275	0.309	0.597
C12:0	0.264	0.301	0.326	0.397	0.386	0.079	0.354	0.456	0.933
C14:0	1.168	1.700	1.710	1.385	1.194	0.499	0.641	0.007	0.037
C16:0	11.702	9.259	8.962	8.970	9.063	1.063	0.073	0.005	0.605
C18:0	3.748	3.242	2.865	2.577	3.135	0.901	0.517	0.857	0.778
C18:1 t11, TVA	0.511^b^	0.747^a^	0.752^a^	0.725^a^	0.751^a^	0.034	<.001	0.483	0.910
C18:1 *c*9	40.685^b^	43.149^ab^	44.418^a^	44.970^a^	44.547^a^	1.624	0.014	0.772	0.798
Σ C18:2 *trans*	0.659	0.371	0.357	0.436	0.385	0.125	0.307	0.493	0.007
C18:2 c9 t11, CLA	0.110	0.114	0.152	0.211	0.156	0.043	0.238	0.145	0.090
C18:2 *c*9, *c*12	30.074	30.415	28.873	28.203	28.798	0.811	0.331	0.034	0.973
C18:3 *c*9, *c*12, *c*15	0.346	0.255	0.203	0.228	0.198	0.051	0.352	0.842	0.863
SFA	20.216^a^	18.248^ab^	17.657^b^	17.769^b^	18.305^ab^	1.141	0.026	0.516	0.486
MUFA	40.737^b^	43.237^ab^	44.477^a^	45.009^a^	44.610^a^	1.656	0.014	0.725	0.801
PUFA	30.550	30.778	29.112	28.512	29.007	0.845	0.292	0.041	0.985

C: control diet; SF60: control diet with sunflower oil added until reaching 60g/kg EE of diet DM; SF75: control diet with sunflower oil added until reaching 75g/kg EE of diet DM; SB60: control diet with soybean oil added until reaching 60g/kg EE of diet DM; SB75: control diet with soybean oil added until reaching 75g/kg EE of diet DM; C10:0: capric acid; C12:0: lauric acid; C14:0: myristic acid; C16:0: palmitic acid; C18:0: stearic acid, C18:1 *t*11, TVA: *trans*-vaccenic acid, C18:1 *c9*: oleic acid; C18:2 *c*9, *t*11, CLA: rumenic acid; C18:2 *c*9 *c*12: linoleic acid; C18:3 *c9, c12, c15*: α-linolenic acid; SFA: saturated fatty acids; MUFA: monounsaturated fatty acids; PUFA: polyunsaturated fatty acids. Values are presented as least-squares means. Means within a row with different superscript letters differ significantly (*P* < 0.05; Tukey–Kramer adjustment). For variables analyzed using PROC GLIMMIX assuming a gamma distribution with log link (C16:0, Σ C18:2 trans, CLA, and C18:3 *c*9, *c*12, *c*15), least-squares means are presented on the original scale using the inverse-link transformation; SEM represents the approximate back-transformed standard error. Covariance structures used for repeated measurements were TOEP for C10:0, C18:2 c9,c12, and PUFA; VC for C12:0 and SFA; UN for C14:0, C16:0, and C18:0; AR(1) for C18:1 t11 (TVA), C18:1 c9, Σ C18:2 trans, and MUFA; CS for CLA; and ARH(1) for C18:3 c9,c12,c15.

The concentration of TVA was lower in the control treatment (least-squares mean: 0.511 g/100 g fat; 95% confidence interval (CI): 0.441–0.582) than in all oil-supplemented treatments (Figure 2). Among the oil-supplemented treatments, least-squares means ranged from 0.725 to 0.752 g/100 g fat (SF60: 0.747, 95% CI: 0.676–0.817; SF75: 0.752, 95% CI: 0.681–0.823; SB60: 0.725, 95% CI: 0.654–0.795; and SB75: 0.751, 95% CI: 0.680–0.821; *P* < 0.001).

**Figure 2:**
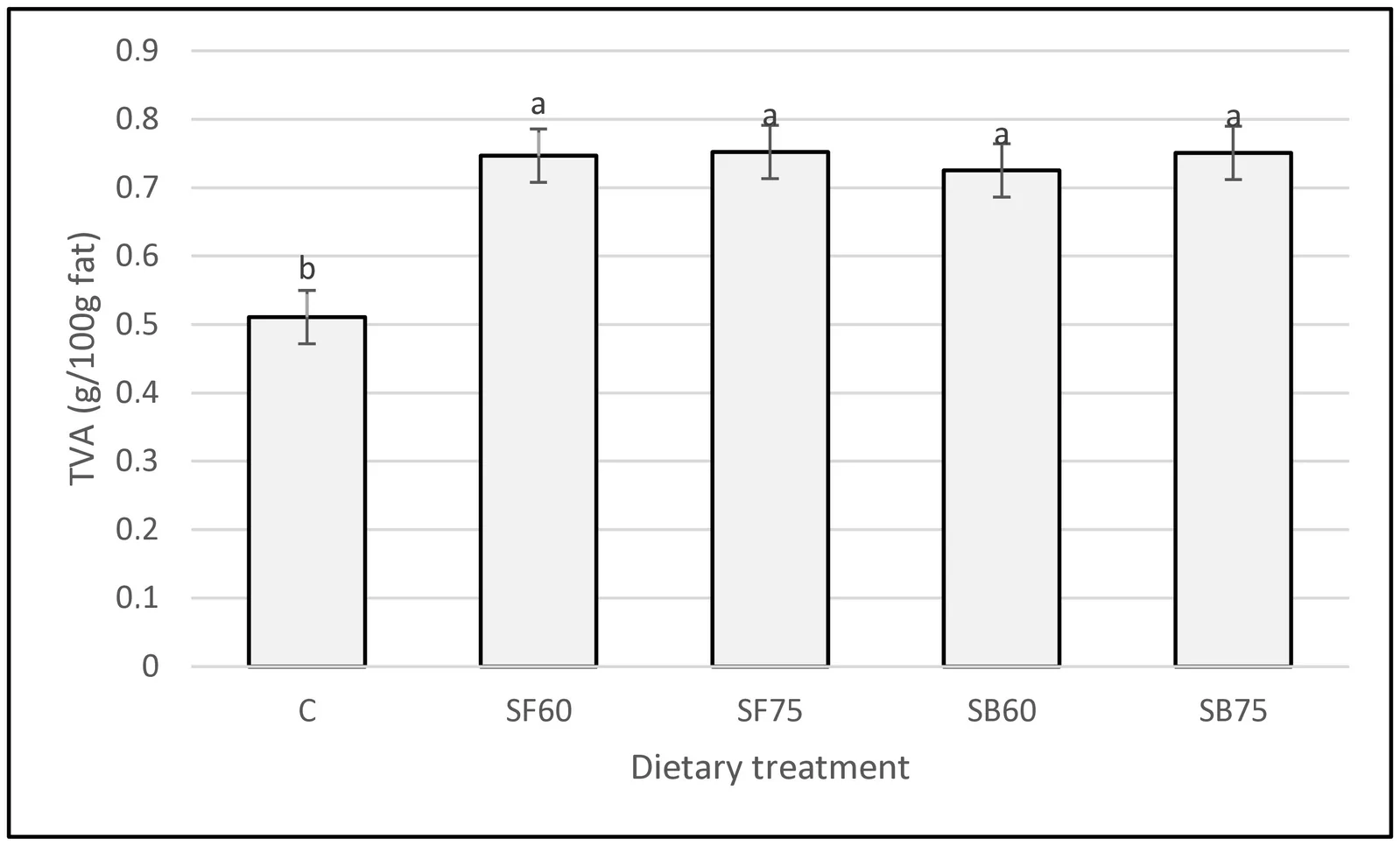
Effects of sunflower and soybean oil supplementation on ruminal trans-vaccenic acid (TVA) concentrations. Bars represent least-squares means, and error bars indicate the SEM. Different letters indicate significant differences among treatments (Tukey–Kramer adjusted, P<0.05). C: control diet; SF60 and SF75: diets containing sunflower oil to achieve 60 and 75 g/kg DM of ether extract, respectively; SB60 and SB75: diets containing soybean oil to achieve 60 and 75 g/kg DM of ether extract, respectively.

The concentration of oleic acid was higher in SF75, SB60, and SB75 than in the control treatment, whereas SF60 showed intermediate values. SFA concentration was lower in SF75 and SB60 than in the control treatment, whereas SF60 and SB75 were intermediate. MUFA concentration was higher in SF75, SB60, and SB75 than in the control treatment, whereas SF60 showed intermediate values.

Sampling day affected the concentrations of myristic acid (C14:0; *P* = 0.007), palmitic acid (C16:0; *P* = 0.005), linoleic acid (C18:2 *c*9, *c*12; *P* = 0.034), and PUFA (*P* = 0.041). Significant treatment × day interactions were detected for C14:0 (*P* = 0.037) and total C18:2 *trans* (*P* = 0.007), whereas a tendency for interaction was observed for rumenic acid (CLA; *P* = 0.090).

## Discussion

Although it is well known that lipid supplementation, especially lipids with a high degree of unsaturation, can affect fermentation and feed degradation in the rumen due to toxic effects on rumen microorganisms (Ferreira et al., 2019; Rabiee et al., 2012), in the present study, no differences were observed among treatments for ruminal pH, gas production, total VFA concentration, or nutrient disappearance. However, oil addition affected ${\text{NH}}_{3}\text{-N}$ concentration, but the response was not consistent across oil sources or inclusion levels. Only SF75 differed from SF60, whereas all other treatments showed intermediate values. Moreover, no differences were observed in nutrient disappearance, suggesting that this was an isolated response rather than a biological effect on ruminal nitrogen metabolism. This interpretation is further supported by the tendency for a sampling day effect on ${\text{NH}}_{3}\text{-N}$ concentration.

The absence of differences among diets in gas production may indicate that fibrolytic bacteria were not affected by oil supplementation, which is consistent with previous studies under similar *in vitro* conditions. Vargas et al. (2019), using sunflower oil added at 6% to a high-concentrate diet, reported that oil addition did not affect *Fibrobacter succinogenes*, while Jalč et al. (2006), using different blends of vegetable oils at 5% added to a high-forage diet, did not observe alterations in ruminal pH, VFA, or DM degradability. In our study, the minimal variations in pH at the Rusitec fermenter could be due to the continuous infusion of artificial saliva maintaining a buffered medium, as reported by Szumacher-Strabel et al. (2004). In addition, a highly buffered medium like this can mask differences between treatments

Total VFA concentration was not affected by the type and level of added oil, which is similar to the results reported by Jacob et al. (2012), who worked with sunflower and soybean oil supplementation at 4% and 6%. In our study, VFA levels ranged from 35.07 to 37.87 mM, values that are relatively low due to the forage-based nature of the experimental diets. Additionally, the meta-analysis by Hristov et al. (2012) reported that VFA values are commonly lower in the Rusitec system when compared with *in vivo* studies, which may also explain our low values.

High levels of oil supplementation typically result in a depression in DM degradation, which was not observed in the present study. Bateman & Jenkins (1998) also reported no effects on ruminal digestibility after supplementing a high-fibrous diet (50% bermudagrass hay and 50% concentrate) with 8% soybean oil, suggesting that fiber creates an environment beneficial to the microorganisms responsible for the hydrolysis and biohydrogenation of dietary fat.

Regarding the fatty acid profile of the ruminal fluid, the main effect of oil supplementation was the increase in TVA concentration. All oil-supplemented diets increased TVA compared with the control diet, indicating that the addition of unsaturated vegetable oils modified ruminal biohydrogenation (BH). This finding is consistent with the classical ruminal BH pathway of linoleic acid (LA). During this process, LA is first isomerized to CLA (mainly the *c*9, *t*11 isomer), subsequently reduced to TVA, and finally hydrogenated to stearic acid (C18:0). Therefore, the greater accumulation of TVA observed in the supplemental treatments suggests that the increased supply of unsaturated fatty acids enhanced the formation of BH intermediates, whereas the final reduction of TVA to stearic acid may have been partially inhibited.

Several ruminal bacterial species participate in the BH of unsaturated fatty acids, especially members of the *Butyrivibrio* genus. During the initial stages of BH, *Butyrivibrio fibrisolvens* plays an important role in catalyzing the isomerization of LA to CLA and the subsequent formation of TVA. In contrast, the final reduction of TVA to stearic acid is mainly attributed to *Butyrivibrio proteoclasticus*. Previous studies have shown that this bacterial species is more susceptible to the inhibitory effects of high concentrations of PUFA, which may reduce its ability to hydrogenate TVA to C18:0. Consequently, inhibition of this terminal step of BH has been proposed as one of the main mechanisms responsible for the accumulation of TVA in diets supplemented with unsaturated vegetable oils. The similar TVA concentrations observed among all oil-supplemented treatments suggest that, under the conditions of the present study, both oil sources and inclusion levels were sufficient to promote a comparable shift in ruminal BH. Despite differences in fatty acid composition between sunflower and soybean oil, both lipid sources produced similar responses in ruminal fatty acid metabolism. Sunflower oil contains a greater proportion of linoleic acid, whereas soybean oil provides both LA and ALA. Nevertheless, both fatty acids undergo ruminal BH and generate TVA as a common intermediate. Therefore, under the conditions of the present study, the total supply of unsaturated fatty acids appeared to have a greater influence on ruminal biohydrogenation than the specific fatty acid composition of each oil.

In contrast, rumenic CLA concentration was not affected by oil supplementation. This finding is not unexpected because only small amounts of CLA are synthesized in the rumen during the isomerization of LA, whereas the majority is synthesized in the mammary gland of ruminants from TVA (Buccioni et al., 2012; Bichi et al., 2012). During milk fat synthesis, the enzyme ${\text{Δ}}^{9}$-desaturase introduces a cis double bond between carbons 9 and 10 of TVA, converting it into rumenic CLA (Palmiquist et al., 2005). Consequently, although CLA concentration in the ruminal fluid was not altered, the greater TVA concentration observed in all oil-supplemented treatments indicates a greater availability of the primary precursor for endogenous CLA synthesis in the mammary gland, where most CLA in milk is produced (Griinari et al., 2000).

Similar results were found by Vargas et al. (2019), who evaluated the *in vitro* effect of supplementation with different vegetable oils (linseed, olive, and sunflower) and observed that diets supplemented with sunflower and linseed oil showed an increase in TVA. Likewise, Jacob et al. (2012) reported increased TVA concentrations following sunflower and soybean oil supplementation. However, unlike the present study, they also observed reductions in ruminal fermentation and nutrient degradability when oils were included at 6% of dietary DM.

The concentration of oleic acid (C18:1 *c*9) was higher in SF75, SB60, and SB75 than in the control treatment, whereas SF60 showed intermediate values. This response may reflect the greater supply of unsaturated fatty acids provided by the vegetable oils, contributing to a shift in the ruminal fatty acid profile toward a higher proportion of monounsaturated fatty acids.

Consistent with the responses observed for oleic acid and TVA, MUFA concentration was higher in SF75, SB60, and SB75 than in the control treatment, whereas SF60 showed intermediate values. Conversely, SFA concentration was lower in SF75 and SB60, while SF60 and SB75 showed intermediate values. Together, these findings demonstrate that vegetable oil supplementation modified ruminal lipid metabolism by shifting the fatty acid profile toward a less saturated composition without affecting overall ruminal fermentation.

Although the Rusitec system provides a controlled environment for evaluating ruminal fermentation and biohydrogenation processes, it has some inherent limitations. The data reported in this study should be interpreted with the understanding that a Rusitec system is used to simulate the rumen, not the total digestive tract, and therefore does not account for nutrient absorption, mammary gland metabolism, or animal-to-animal variation (Hristov et al., 2012). In addition, as commonly occurs in Rusitec experiments, the number of experimental units was constrained by the capacity of the fermentation system. Each treatment was evaluated in three independent experimental runs, providing three experimental units per treatment. Although this balanced randomized block design allowed robust comparisons among treatments under controlled experimental conditions, the relatively limited replication may have reduced the ability to detect small biological effects, particularly for variables with greater variability. Nevertheless, the Rusitec system remains a valuable experimental model because it allows highly controlled evaluation of ruminal fermentation processes under standardized laboratory conditions while substantially reducing the need for live animals. The observed increases in TVA demonstrate the potential of soybean and sunflower oil supplementation to modify ruminal lipid metabolism under controlled ruminal conditions. These findings provide important information about ruminal biohydrogenation and establish a solid basis for future *in vivo* studies to determine how these changes are translated into the fatty acid composition of milk and meat.

## Conclusion

Sunflower and soybean oil supplementation effectively increased TVA concentration in the ruminal fluid without negatively affecting ruminal fermentation or nutrient disappearance. These findings indicate that both vegetable oils are effective in modifying ruminal biohydrogenation processes and provide a basis for future *in vivo* studies to determine whether these changes are translated into improvements in the fatty acid composition of milk and meat.
